# In Vitro Evaluation of the Antifungal Activity and Cytotoxicity of *Sambucus williamsii* var. *coreana* Extract Against *Candida albicans* and HaCaT Cells

**DOI:** 10.3390/pathogens15060635

**Published:** 2026-06-15

**Authors:** Hyo-Ju Yoon, Gyoo-Cheon Kim, Seoul-Hee Nam

**Affiliations:** 1 Department of Dental Hygiene, College of Health Sciences, Kangwon National University, Samcheok-si 25949, Republic of Korea; cloven79@naver.com; 2 Department of Oral Anatomy, School of Dentistry, Pusan National University, Yangsan-si 50612, Republic of Korea

**Keywords:** *Sambucus williamsii* var. *coreana*, *Candida albicans*, antifungal activity, cytotoxicity, HaCaT cells

## Abstract

Background: Oral candidiasis is an opportunistic infection caused by *Candida albicans (C. albicans)*, highlighting the need to evaluate candidate substances that ensure both antifungal efficacy and mucosal safety. This study aimed to assess the potential of *Sambucus williamsii* var. *coreana* (*S. williamsii* var. *coreana*) extract as a naturally derived antifungal agent for topical oral application by investigating its antifungal activity against *C. albicans* and its cytotoxicity in human keratinocyte (HaCaT) cells. Methods: *S. williamsii* var. *coreana* was extracted with 70% ethanol, concentrated, and freeze-dried. The extract was prepared at concentrations of 1–40 mg/mL and applied under 6 h and 24 h exposure conditions. Antifungal activity against cultured *C. albicans* was evaluated using colony-forming unit (CFU) analysis, while cytotoxicity in HaCaT cells was assessed via the Water-soluble Tetrazolium Salt-1 assay after incubation at 37 °C in 5% CO_2_ for 2 h. Statistical significance was analyzed using Student’s *t*-test, ANOVA, and Tukey’s HSD test (*p* < 0.05). Results: The *S. williamsii* var. *coreana* extract exhibited concentration- and time-dependent antifungal activity. A 99.99% inhibition of *C. albicans* was observed at 5 mg/mL. No detectable CFUs were observed at 30 mg/mL after 6 h and at 10 mg/mL after 24 h. HaCaT cell viability decreased in a concentration-dependent manner, with the half-maximal inhibitory concentration determined to be 10 mg/mL. Conclusions: The extract of *S. williamsii* var. *coreana* exhibited concentration- and time-dependent in vitro antifungal activity against *C. albicans*. However, the concentration associated with no detectable CFUs overlapped with the cytotoxic concentration range in HaCaT cells, indicating that further studies are required to define an appropriate concentration range for potential oral application.

## 1. Introduction

*Candida* species are major opportunistic fungal pathogens that commonly colonize mucosal surfaces, including the oral cavity, gastrointestinal tract, and vagina, and may also be detected in the oral cavity of healthy individuals [1]. Among them, *Candida albicans* (*C. albicans*) is the most prevalent pathogenic species in the oral cavity and is a principal causative organism of oral candidiasis [2]. The pathogenicity of *C. albicans* is associated with morphological switching between yeast and hyphal forms, adhesion to host epithelial cells, tissue invasion, and biofilm formation, which contribute to persistence and treatment resistance [2,3]. Previous antifungal exposure may be associated with changes in *Candida* species distribution and antifungal susceptibility, highlighting the need for alternative antifungal candidates [4]. Oral candidiasis is closely associated with immunosuppression, xerostomia, denture use, antibiotic exposure, and poor oral hygiene, conditions under which *C. albicans* may shift from commensal colonization to pathogenic overgrowth on oral mucosal or denture surfaces [3,5,6]. Therefore, antifungal strategies that suppress pathogenic fungal proliferation while considering mucosal safety are necessary for the prevention and management of oral candidiasis [3,6]. The oral cavity is a complex ecosystem in which diverse microorganisms coexist, and maintaining microbial balance is crucial for oral health. However, excessive use of antibiotics, antimicrobial agents, or disinfectants can disrupt this ecological balance, leading to microbiome dysbiosis and increased susceptibility to opportunistic infections [7]. The oral microbiome comprises bacterial, fungal, viral, and other microbial communities that interact with host tissues and contribute to oral homeostasis [8]. In addition to *C. albicans*, non-*albicans Candida* species, including *C. glabrata*, *C. tropicalis*, *C. krusei*, and *C. parapsilosis*, may also be detected in the oral cavity and can contribute to oral fungal infections under susceptible host conditions [9].

Oral candidiasis often responds inadequately to treatment or recurs frequently, and repeated use of conventional antifungal agents raises concerns regarding reduced drug susceptibility, emergence of azole-resistant strains, and host cytotoxicity [3,6]. Particularly for agents applied directly to the oral mucosa, it is necessary to establish a concentration range that provides antifungal efficacy while minimizing epithelial cytotoxicity. Accordingly, increasing attention has recently been directed toward naturally derived antimicrobial agents as alternatives or complementary materials to synthetic antifungal agents. Natural compounds may exhibit antimicrobial activity with relatively favorable epithelial compatibility at appropriate concentrations, thereby supporting continued research on natural antifungal candidates applicable to the oral environment [10,11].

*Sambucus williamsii* var. *coreana* (*S. williamsii* var. *coreana*) is a traditionally used medicinal plant with reported biological activities, including anti-inflammatory, antioxidant, and bone metabolism-regulating effects [12]. This plant was selected as a candidate material because previous studies have reported biological activities of *S. williamsii* and antifungal activity of its isolated lignan compound, (+)-pinoresinol [12,13]. In particular, (+)-pinoresinol, a lignan isolated from *S. williamsii*, has demonstrated antifungal activity against *C. albicans*, and a mechanism involving cell membrane disruption has been proposed [13]. Although the presence or concentration of (+)-pinoresinol was not determined in the crude extract used in the present study, these findings provide a rationale for further evaluating *S. williamsii* var. *coreana* as a source of naturally derived antifungal candidates. However, studies simultaneously evaluating its antifungal activity against oral pathogenic fungi and its effects on epithelial cell viability remain limited.

Keratinocytes are the predominant epithelial cells of the oral mucosa and contribute to epithelial barrier integrity, host–microbe interactions, and local innate immune responses [14]. HaCaT cells are an immortalized human keratinocyte cell line that retains the growth and differentiation characteristics of epithelial cells, making them widely used to evaluate epithelial cell responses and cytotoxicity [15]. However, the relationship between the effects of *S. williamsii* var. *coreana* extract on HaCaT cell viability and its antifungal concentration range against *C. albicans* has not been fully elucidated. Furthermore, the balance between efficacy and safety—specifically, the extent to which antifungal concentrations overlap with cytotoxic concentration ranges in epithelial cells—is a key factor in evaluating its potential oral applicability.

Accordingly, this study aimed to evaluate the concentration- and time-dependent in vitro antifungal activity of *S. williamsii* var. *coreana* extract against *C. albicans* and to examine its cytotoxic effects on HaCaT cells under the same exposure conditions. This study also sought to provide preliminary evidence for its potential development as a naturally derived antifungal material for the prevention and management of oral diseases.

## 2. Materials and Methods

### 2.1. Extraction Process of S. williamsii var. coreana

The stems of *S. williamsii* var. *coreana* were procured from Cheongmyeong Co., Ltd. (Goesan, Chungcheongbuk-do, Republic of Korea). The material was ground and extracted with 70% ethanol at 60 °C for 12 h and then filtered. The extract was concentrated using a rotary vacuum evaporator (N-1300E.V.S. EYELA, Rikakikai Co., Ltd., Tokyo, Japan). Finally, the concentrate was freeze-dried at −80 °C using a freeze dryer (Ilshin Lab Co., Yangju-si, Republic of Korea) to obtain a powdered sample.

### 2.2. Strain Preparation and Cultivation Conditions

*C. albicans* (KCTC 27242) was obtained from the American Type Culture Collection (Manassas, VA, USA) and inoculated into yeast mold (YM) broth (Difco, Detroit, MI, USA). After two successive subcultures, the strain was incubated at 37 °C for 24 h to ensure adequate growth and maintain metabolic activity.

### 2.3. Effect of S. williamsii var. coreana Extract Against C. albicans

To evaluate the antifungal activity of the *S. williamsii* var. *coreana* extract, viable cell counts were determined against *C. albicans*. *C. albicans* cultures were adjusted to an initial inoculum density of approximately 1 × 10^6^ CFU/mL. A total volume of 1 mL was prepared by mixing 900 μL of the culture suspension with 100 μL of *S. williamsii* var. *coreana* extract at concentrations of 1, 3, 5, 10, 20, 30, and 40 mg/mL. After treatment, the suspension was serially diluted tenfold, and 1 mL of each culture suspension was plated onto YM agar medium. Following aerobic incubation at 37 °C, colony-forming units (CFUs) were counted after 6 and 24 h to quantitatively assess the concentration-dependent antifungal activity of the extract. All experiments were conducted independently in triplicate, and the data are expressed as the mean ± standard deviation (SD).

### 2.4. Assessment of Cytotoxicity Using Water-Soluble Tetrazolium Salt-1

Cell viability and proliferation were quantitatively evaluated using the EZ-Cytox Cell Viability Assay Kit (ITSBIO Inc., Seoul, Republic of Korea), based on the Water-soluble Tetrazolium Salt (WST-1) assay principle [16]. HaCaT cells, an established immortalized human keratinocyte cell line, were obtained from the Department of Oral Anatomy, School of Dentistry, Pusan National University (Yangsan, Republic of Korea). HaCaT cells were cultured at 37 °C in 5% carbon dioxide (CO_2_) using Dulbecco’s Modified Eagle’s Medium (Gibco, Grand Island, NY, USA) supplemented with 10% fetal bovine serum (Gibco, Grand Island, NY, USA) and 1% penicillin–streptomycin (Gibco, Grand Island, NY, USA). HaCaT cells were seeded into 96-well plates at a density of 1 × 10^5^ cells/well in 100 µL medium and incubated at 37 °C for 24 h. The cells were then treated with extract concentrations of 1, 3, 5, 10, 20, 30, and 40 mg/mL for 6 and 24 h. Subsequently, 10 µL of kit reagent was added to each well, followed by incubation for 2 h at 37 °C in 5% CO_2_. Absorbance was measured at 450 nm using an enzyme-linked immunosorbent assay reader (Multiskan FC, Thermo Fisher Scientific, Waltham, MA, USA). All experiments were independently repeated at least three times, and the results are presented as mean ± SD.

### 2.5. Statistical Analysis

Statistical analyses were performed using SPSS software (version 24.0; SPSS Inc., Chicago, IL, USA). Time-dependent differences were evaluated using Student’s *t*-test, whereas concentration-dependent differences were analyzed using one-way analysis of variance (ANOVA) followed by Tukey’s honest significant difference (HSD) test. Statistical significance was set at *p* < 0.05.

## 3. Results

### 3.1. Concentration-Dependent Antifungal Activity

**Figure 1:**
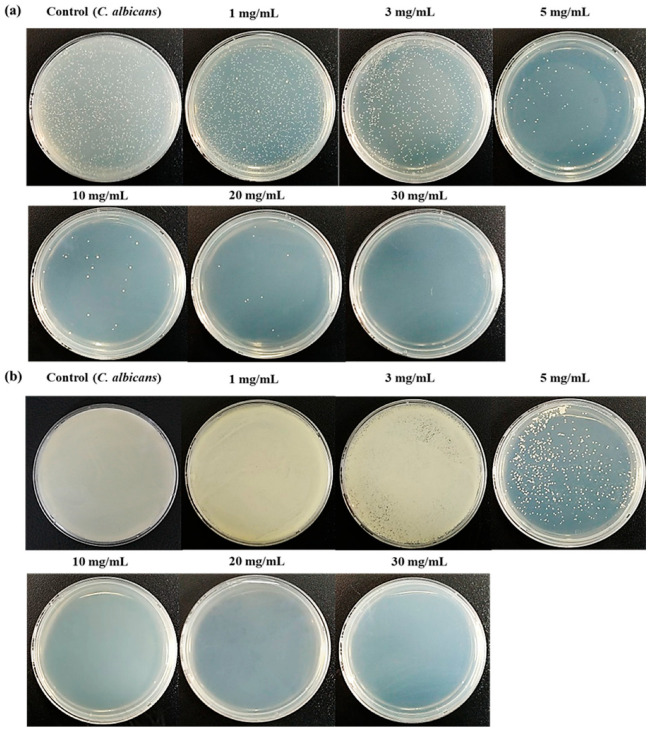
Antifungal activity of *S. williamsii* var. *coreana* extract against *C. albicans* after (**a**) 6 h and (**b**) 24 h.

**Figure 2:**
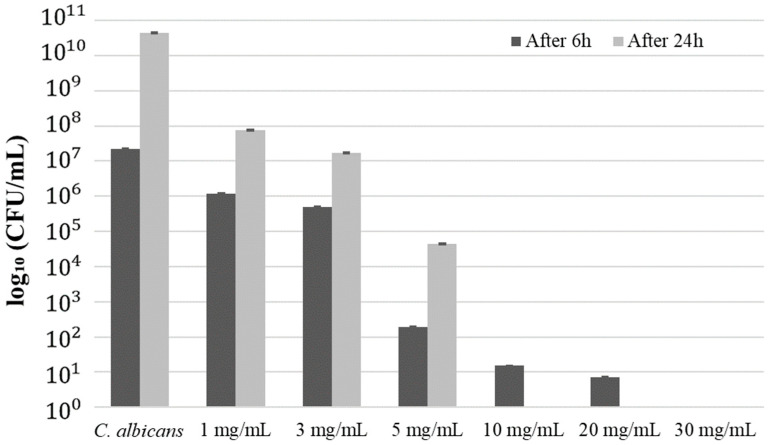
Changes in colony-forming unit counts (log_10_ CFU/mL) of *C. albicans* after treatment with *S. williamsii* var. *coreana* extract for 6 and 24 h. Data are expressed as the mean ± standard deviation (SD) of three independent experiments. Error bars indicate SD.

*S. williamsii* var. *coreana* exhibited concentration- and time-dependent antifungal activity against *C. albicans*. The inhibitory effect increased with rising extract concentration (Figure 1). Antifungal activity, determined by CFU counts after 6 and 24 h, is presented in Figure 2. The extract-treated groups showed a significant reduction in CFU counts as concentration increased, and this trend was observed at both time points. These findings indicate that inhibition of fungal growth became more pronounced with increasing concentration and exposure time. Notably, no CFUs were detected in the 30 mg/mL treatment group after 6 h or in the 10 mg/mL treatment group after 24 h, indicating complete suppression of *C. albicans* proliferation under these conditions.

As shown in Table 1, Student’s *t*-test demonstrated significant differences between the 6 and 24 h treatment periods at the same concentration (*p* < 0.05). CFU counts were significantly lower after 24 h than after 6 h, confirming enhanced antifungal activity over time. At 30 mg/mL, no viable colonies were detected at either time point, indicating complete fungal eradication.

When concentrations were compared at each time point, significant differences between groups were identified using one-way ANOVA and Tukey’s HSD post hoc test (*p* < 0.05). After 6 h of treatment, fungal survival was significantly reduced compared with the control group at concentrations of 1 mg/mL or higher, and CFU counts progressively decreased as the concentration increased. At 5 mg/mL, 99.99% inhibition was observed compared to the control group, whereas no CFUs were detected at 30 mg/mL, indicating complete antifungal activity. After 24 h of treatment, the antifungal activity was greater than that observed after 6 h. At 1 mg/mL, 99.82% inhibition was observed compared with the control group, while 5 mg/mL resulted in 99.99% inhibition. Starting from 10 mg/mL, almost no CFUs were detected, indicating complete fungal inactivation. In addition, within the concentration range of 5–30 mg/mL, no significant differences were observed among treatment groups at either time point, suggesting consistently strong antifungal activity across this range.

### 3.2. Toxic Effects on Cell Viability

**Figure 3:**
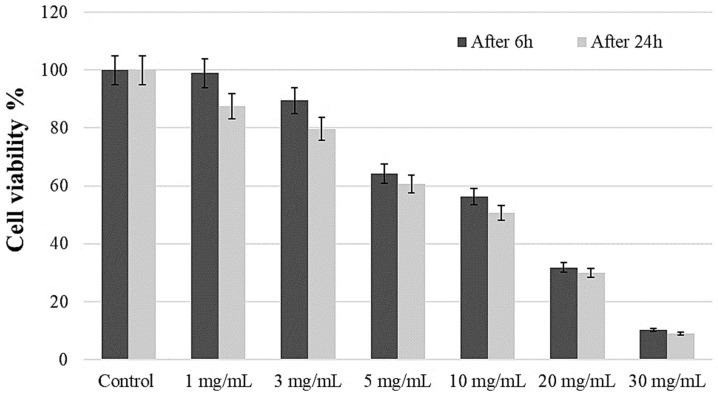
Cell viability of immortalized HaCaT cells treated with *S. williamsii* var. *coreana* extract, as determined by the WST-1 assay. Data are expressed as the mean ± standard deviation (SD) of three independent experiments. Error bars indicate SD.

Figure 3 presents the WST-1 assay results for the cytotoxic effects of *S. williamsii* var. *coreana* extract on HaCaT cells according to concentration and exposure time. Cell viability decreased markedly with increasing concentration, indicating that the cytotoxic effect of the extract was more strongly influenced by concentration than by exposure.

The half maximal inhibitory concentration (IC_50_) was determined to be 10 mg/mL. At this concentration, cell viability was 56% after 6 h of treatment and 50% after 24 h (Table 2). Cell viability declined significantly as the concentration increased (*p* < 0.05), and significant differences were also observed between exposure times at the same concentration, except in the control group (*p* < 0.05). Notably, inhibition of HaCaT cell proliferation became evident at concentrations of 20 mg/mL or higher, indicating increased cytotoxicity at elevated doses. The 10 mg/mL concentration may represent a threshold at which moderate cell viability is maintained without rapid loss of cells.

## 4. Discussion

Oral candidiasis is an opportunistic infection caused by interactions between host immune status and fungal virulence factors. It is closely associated with acquired immunodeficiency syndrome, diabetes, and immunosuppression, and often presents with a recurrent or chronic clinical course [3]. These characteristics indicate the need for antifungal candidates that can inhibit fungal growth while minimizing epithelial cytotoxicity. Previous studies have reported that treatment with 0.12% chlorhexidine, which is widely used in clinical practice, reduces HaCaT cell viability to approximately 40–50% [17]. Therefore, naturally derived antimicrobial materials should be evaluated for both antifungal activity and epithelial cell compatibility within an appropriate concentration range.

The oral cavity is a complex ecosystem in which diverse microorganisms, including bacteria, fungi, and viruses, coexist. Oral microbial homeostasis is maintained through dynamic interactions among these communities. Under normal conditions, commensal microorganisms help suppress the excessive proliferation of pathogenic species [8]. However, infectious oral diseases such as oral candidiasis may involve mixed bacterial–fungal biofilms, which can contribute to recurrence after treatment or reduced therapeutic response [18]. Several limitations of currently used antifungal agents, including side effects, drug interactions, and toxicity, have been reported [19]. In particular, the oral mucosa is continuously exposed to external stimuli and serves as a first-line barrier against direct contact with *Candida*. Therefore, antifungal candidates intended for oral application should be evaluated not only for their inhibitory effects on pathogenic fungi but also for their safety toward epithelial cells within a clinically relevant concentration range [20]. Accordingly, this study evaluated the antifungal activity of *S. williamsii* var. *coreana* extract against *C. albicans* and examined its effects on HaCaT cell viability and toxicity. The aim was to identify a natural antifungal candidate capable of suppressing oral pathogenic fungi while minimizing toxicity to oral epithelial cells.

The results of this study demonstrated that S. williamsii var. coreana extract reduced viable *C. albicans* counts in a concentration- and time-dependent manner. After 6 h of treatment, a 94.78% reduction relative to the control group was observed at 1 mg/mL, while 99.99% inhibition was observed at 5 mg/mL. No detectable CFUs were observed at 30 mg/mL. After 24 h, the antifungal response increased further, with 99.82% inhibition at 1 mg/mL and 99.99% inhibition at 5 mg/mL. No detectable CFUs were observed at 10 mg/mL.

These findings indicate that the extract markedly reduced *C. albicans* proliferation under the tested in vitro conditions. The greater CFU reduction after 24 h than after 6 h suggests that exposure duration contributed to the antifungal response. However, because cytotoxicity increased with concentration, the effective antifungal concentration range should be interpreted together with the corresponding effects on HaCaT cell viability.

These findings are comparable to those reported by Kim et al. [21], who found that *Chamaecyparis obtusa* extract showed concentration-dependent antifungal activity against the same strain of *C. albicans*. The present results showed a similar concentration-dependent pattern and further suggest that exposure time may influence the antifungal response. However, direct comparison between studies should be interpreted cautiously because differences in extract composition, experimental conditions, and cytotoxicity profiles may affect the observed antifungal activity.

For antifungal candidates intended for the management of oral infectious diseases, it is essential to ensure antifungal efficacy while establishing an appropriate concentration range that considers epithelial cell viability for potential oral application. In this study, cytotoxicity was evaluated under the same exposure conditions used for antifungal testing by assessing HaCaT cell viability and the IC_50_ value. The extract caused a concentration-dependent decrease in cell viability. At the IC_50_ of 10 mg/mL, HaCaT cell viability was 56% after 6 h and 50% after 24 h. Notably, no detectable *C. albicans* CFUs were observed at the same concentration after 24 h. This overlap indicates that the concentration associated with strong antifungal activity also corresponded to a cytotoxic range in HaCaT cells. Therefore, antifungal activity should be interpreted together with epithelial cell viability when defining an appropriate concentration range for further oral application studies.

Previous studies have evaluated the applicability of natural extracts using HaCaT cells by comparing antifungal efficacy with HaCaT cell viability. One study reported that *Chamaecyparis obtusa* extract showed antifungal activity against *C. albicans* at 2.5 mg/mL, with no detectable fungal growth at 30 mg/mL. However, HaCaT cell viability decreased to approximately 61% at 2.5 mg/mL and to 20% at 5 mg/mL, indicating a rapid decline in cell viability within the concentration range associated with increasing antifungal activity [21]. Similarly, *Rhoicissus tridentata* root extract demonstrated antifungal activity at 0.78 mg/mL, while the IC_50_ in HaCaT cells was 121.3 ± 6.0 μg/mL [22]. Although the extract was active at low concentrations, cytotoxicity also appeared within a relatively low dose range. In addition, *Rhus verniciflua Stokes* extract has shown more than 99% inhibition of *C. albicans* growth at 1.25 mg/mL, but HaCaT cell viability at the same concentration was only 17.21% [23]. These findings suggest that antifungal activity alone does not ensure suitability for oral application if HaCaT cell viability is substantially reduced. In contrast, the present study showed that treatment with 10 mg/mL of *S. williamsii* var. *coreana* extract resulted in HaCaT cell viability of 56% after 6 h and 50% after 24 h, while no detectable *C. albicans* CFUs were observed at the same concentration after 24 h. This study is meaningful in that it evaluated antifungal activity and HaCaT cell viability under the same exposure conditions. However, because the concentration at which no detectable CFUs were observed overlapped with the IC_50_ range in HaCaT cells, further studies are required to define an effective concentration range with improved epithelial compatibility.

This study was conducted under in vitro conditions using a single planktonic strain of *C. albicans*. Since the oral cavity is a complex environment in which pathogenicity is influenced by saliva, host immune responses, interactions among diverse microorganisms, and biofilm formation, the antifungal activity observed under simplified culture conditions may not fully reflect the actual oral environment [8,18,24]. In addition, phytochemical characterization and standardization of the extract were not performed, and conventional antifungal agents were not included as positive controls, limiting direct comparison with clinically used agents and interpretation of the compounds responsible for the observed activity. Because the epithelial cell compatibility findings were based solely on in vitro assays, they cannot be directly extrapolated to clinical conditions. Future studies should therefore include saliva-containing experimental conditions, biofilm models, additional clinical or resistant strains, non-*albicans Candida* species, standardized MIC/MFC assays, and comparative evaluations with reference antifungal agents. Moreover, molecular-level analyses, including identification of major bioactive substances, membrane integrity, ROS generation, apoptosis/necrosis, and fungal morphological changes, are needed to clarify the underlying mechanisms of antifungal activity and cytotoxicity.

Overall, the present findings provide an integrated view of the antifungal activity of *S. williamsii* var. *coreana* extract and its effects on HaCaT cell viability under in vitro conditions. The CFU results indicate that the extract markedly reduced viable fungal counts, with no detectable CFUs under specific concentration and exposure-time conditions. In addition, HaCaT cell viability and the IC_50_ value provided useful information for interpreting the relationship between antifungal efficacy and epithelial cell response. Notably, the concentration at which no detectable CFUs were observed after 24 h overlapped with the IC_50_ range in HaCaT cells, indicating that cytotoxicity should be carefully considered when defining an effective concentration range in subsequent studies. Therefore, the present findings provide preliminary evidence that *S. williamsii* var. *coreana* extract may be further investigated as a naturally derived antifungal candidate. However, additional studies under more clinically relevant oral conditions are required before its applicability for oral candidiasis management can be established.

## 5. Conclusions

This study evaluated the antifungal activity of *S. williamsii* var. *coreana* extract against *C. albicans* and its cytotoxic effects on HaCaT cells under identical experimental conditions. A 99.99% reduction in CFU counts was observed at 5 mg/mL. No detectable CFUs were observed at 30 mg/mL after 6 h of treatment and at 10 mg/mL after 24 h, demonstrating concentration- and time-dependent antifungal activity. The cytotoxicity IC_50_ was determined to be 10 mg/mL. These findings suggest that *S. williamsii* var. *coreana* extract may be further investigated as a naturally derived antifungal candidate for potential oral application. Further validation is needed to clarify the balance between antifungal efficacy and HaCaT cell viability before proceeding to further oral application studies.

## Figures and Tables

**Table 1:** Antifungal activity of *S. williamsii* var. *coreana* extract against *C. albicans* based on CFU counts according to concentration and exposure time.

Concentration (mg/mL)	Time (h)	log_10_ CFU/mL	*t*-Test *p*-Value	ANOVA *p*-Value
0	6	2.25 ± 0.9 × 10^7 a^	<0.001 *	<0.001 †
	24	4.39 ± 0.5 × 10^10 a^		
1	6	1.18 ± 0.1 × 10^1 b^	<0.001 *	
	24	7.70 ± 0.6 × 10^7 b^		
3	6	5.00 ± 0.8 × 10^1 c^	<0.001 *	
	24	1.69 ± 0.4 × 10^7 c^		
5	6	1.91 ± 0.4 × 10^1 d^	<0.001 *	
	24	4.47 ± 0.9 × 10^4 d^		
10	6	1.50 ± 0.7 × 10^1 d^	<0.001 *	
	24	0.00 ± 00 ^d^		
20	6	7.00 ± 0.8 10^1 d^	<0.009 *	
	24	0.00 ± 00 ^d^		
30	6	0.00 ± 00 ^d^	-	
	24	0.00 ± 00 ^d^		

* Significant difference between the 6 h and 24 h treatment groups, as determined by Student’s *t*-test (*p* < 0.05). † Significant differences among concentrations at each time point were analyzed using one-way analysis of variance. Different letters (a–d) indicate statistically significant differences according to Tukey’s honest significant difference post hoc test (*p* < 0.05).

**Table 2:** Effects of *S. williamsii* var. *coreana* extract on immortalized HaCaT cell viability at different concentrations and time points.

Concentration (mg/mL)	Time (h)	Cell Viability (% of Control)	*t*-Test *p*-Value	ANOVA *p*-Value
0	6	100.0 ± 1.0 ^a^	1.000	<0.001 †
	24	100.0 ± 1.1 ^a^		
1	6	99.0 ± 0.1 ^b^	<0.001 *	
	24	87.5 ± 0.3 ^b^		
3	6	89.5 ± 0.4 ^c^	<0.001 *	
	24	79.4 ± 0.6 ^c^		
5	6	64.3 ± 0.2 ^d^	<0.001 *	
	24	60.7 ± 0.5 ^d^		
10	6	56.3 ± 0.3 ^e^	<0.001 *	
	24	50.8 ± 0.2 ^e^		
20	6	31.9 ± 0.5 ^f^	<0.001 *	
	24	29.9 ± 0.8 ^f^		
30	6	10.2 ± 0.3 ^g^	<0.001 *	
	24	9.1 ± 0.7 ^g^		

* Statistical differences between the 6 h and 24 h groups were evaluated using Student’s *t*-test (*p* < 0.05). † Differences among concentrations at each time point were analyzed using one-way analysis of variance followed by Tukey’s honest significant difference post hoc test. Values with different superscript letters (a–g) indicate statistically significant differences (*p* < 0.05).

## Data Availability

The original contributions presented in this study are included in the article. Further inquiries can be directed to the corresponding authors.
